# Vaginal prolapse with urinary bladder incarceration and consecutive irreducible rectal prolapse in a dog

**DOI:** 10.1186/s13028-016-0235-2

**Published:** 2016-09-22

**Authors:** Ciprian-Andrei Ober, Cosmin Petru Peștean, Lucia Victoria Bel, Marian Taulescu, Cornel Cătoi, Sidonia Bogdan, Joshua Milgram, Guenter Schwarz, Liviu Ioan Oana

**Affiliations:** 1 Department of Surgery, University of Agricultural Sciences and Veterinary Medicine, 3-5 Mănăştur Street, 400372 Cluj-Napoca, Romania; 2 Department of Anesthesiology and Intensive Care, University of Agricultural Sciences and Veterinary Medicine, 3-5 Mănăştur Street, 400372 Cluj-Napoca, Romania; 3 Department of Veterinary Pathology, University of Agricultural Sciences and Veterinary Medicine, 3-5 Mănăştur Street, 400372 Cluj-Napoca, Romania; 4 Veterinary Teaching Hospital, Koret School of Veterinary Medicine, The Hebrew University of Jerusalem, P.O. Box 12, 76100 Rehovot, Israel; 5 Tierklinik Hollabrunn, Lastenstraße 2, 2020 Gemeinde Hollabrunn, Austria

**Keywords:** Dogs, Rectal prolapse, Urinary bladder, Vaginal prolapse

## Abstract

**Background:**

True vaginal prolapse is a rare condition in dogs and it is occasionally observed in animals with constipation, dystocia, or forced separation during breeding. If a true prolapse occurs, the bladder, the uterine body and/or distal part of the colon, may be present in the prolapse.

**Case presentation:**

A 2-year-old intact non pregnant Central Asian Shepherd dog in moderate condition, was presented for a true vaginal and rectal prolapse. The prolapses were confirmed by physical examination and ultrasonography. Herniation of the urinary bladder was identified within the vaginal prolapse. The necrotic vaginal wall was resected, the urinary bladder was reduced surgically and fixed to the right abdominal wall to prevent recurrence. Rectal resection and anastomosis was necessary to correct the rectal prolapse. Recurrence of the prolapses was not observed and the dog recovered completely after the surgical treatment.

**Conclusions:**

In our opinion, extreme tenesmus arising from constipation may have predisposed to the vaginal prolapse with bladder incarceration and secondarily to rectal prolapse. In the young female dog, true vaginal prolapse with secondary involvement of the urinary bladder and irreducible rectal prolapse is an exceptionally rare condition.

## Background

The most common causes of vaginal/vestibular masses in the bitch are vaginal prolapse, vaginal neoplasia, and urethral neoplasia protruding into the vaginal vault [1]. True vaginal prolapse is a rare condition in dogs and cats, but it is occasionally found in animals with constipation, dystocia, or forced separation during breeding [2, 3].

When a true prolapse occurs, other structures such as the bladder, the uterine body and/or the descending colon may be present within the prolapse [4]. In four of five reported canine cases of vaginal prolapse, other structures had herniated into the vagina [2, 3, 5] with development of urinary bladder incarceration in two cases [6, 7].

In case of rectal prolapse, all layers of the rectum protrude through the anal orifice as an elongated, cylindrical mass. Prolapse usually develops secondary to tenesmus from urogenital or anorectal disorders associated with predisposing conditions, including gastrointestinal nematodiasis; typhlitis; colitis; proctitis; tumors of the colon, rectum, or anus; rectal foreign bodies; perineal hernia; cystitis; prostatic disease; urolithiasis; and dystocia [8].

This case report describes a true vaginal prolapse with bladder incarceration combined with an irreducible rectal prolapse in a young, non-pregnant, large breed dog. To our knowledge, only one case of this condition has been reported [7], but the rectal prolapse was reducible in that case.

## Case presentation

**Fig. 1:**
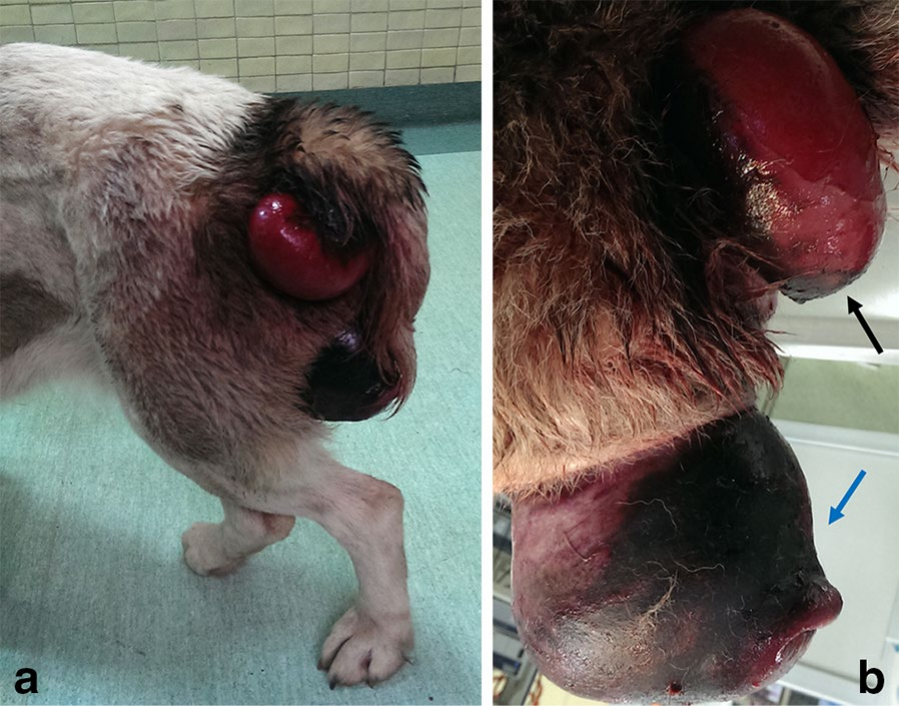
Clinical and pathological features of vaginal and rectal prolapses in a Central Asian Shepherd bitch. **a**, **b** Gross appearance of prolapsed vagina (*blue arrow*) and rectum (*black arrow*). Note the congested, edematous and necrotic appearance of the vaginal mucosa

A 2-year-old intact female Central Asian Shepherd dog in moderate condition, body weight (BW) 42 kg, was presented with obvious masses in the anus and vulva (Fig. 1a). A large regularly shaped mass protruded through the vulvar commissure. The owner explained that the mass had developed gradually during the last 2 months. The mass in the anus had been observed 6 days before the physical examination and had also enlarged gradually. No local or systemic medication had been administered. Straining, dysuria and tenesmus were the main reasons for presentation at the clinic. The owner reported that the dog also showed lethargy and moderately decreased appetite.

During the examination, the dog was not particular sensitive to palpation and manipulation of the prolapsed masses. The temperature, peripheral pulse, and respiratory rate were within normal ranges. Hematology including complete blood count and serum biochemistry profiles. All parameters were within normal range.

**Fig. 2:**
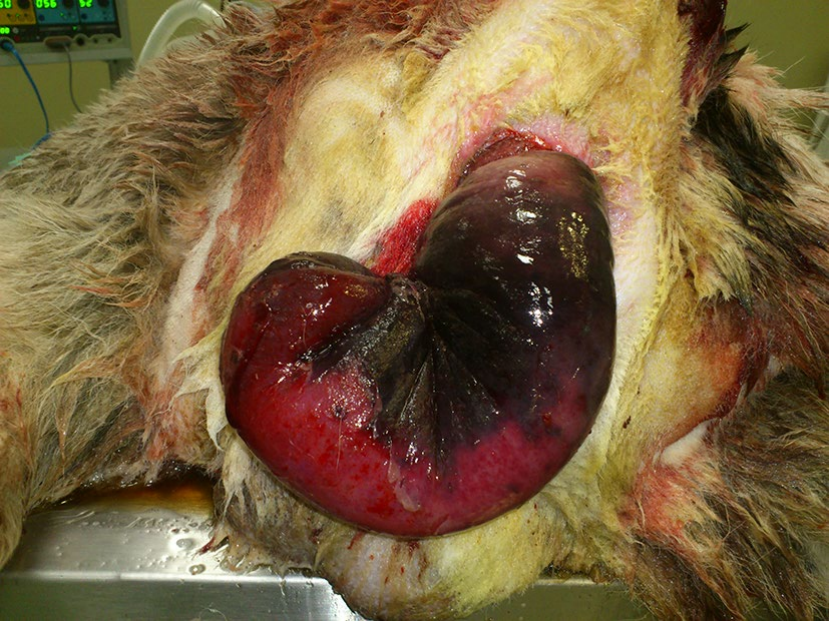
Rectal prolapse with severe thickening of the wall due to congestion and edema and extensive superficial necrosis (*frontal view*)

The protruding vaginal and rectal masses showed extensive congestion, hemorrhages, edema and superficial necrosis (Figs. 1b, 2). On transabdominal ultrasonography, the urinary bladder was not detected in its normal anatomic position, but ultrasonography of the prolapsed vagina revealed a nonechogenic, fluid-filled and bladder-like body inside the prolapse. Needle aspiration of urine from the mass and ultrasonography confirmed retroflexion of the bladder. Consequently, the external urethral orifice could not be catheterized.

The dog was administered cefazolin preoperatively (22 mg/kg BW intravenously; Cefazolin, Sandoz GMBH, Austria). General anesthesia was induced with propofol (4 mg/kg BW; Braun, Melsungen, Germany) and maintained with isoflurane (Forane; Abbott, Romania) delivered in 100 % oxygen. Lumbosacral analgesia was carried out with morphine (0.1 mg/kg BW; Morfină, Sicomed S.A., Bucharest, Romania). Lactated Ringer’s solution was administered during surgery (20 ml/kg BW per hour; Soluție Ringer Lactat, Braun Medical, Timiș, Romania).

**Fig. 3:**
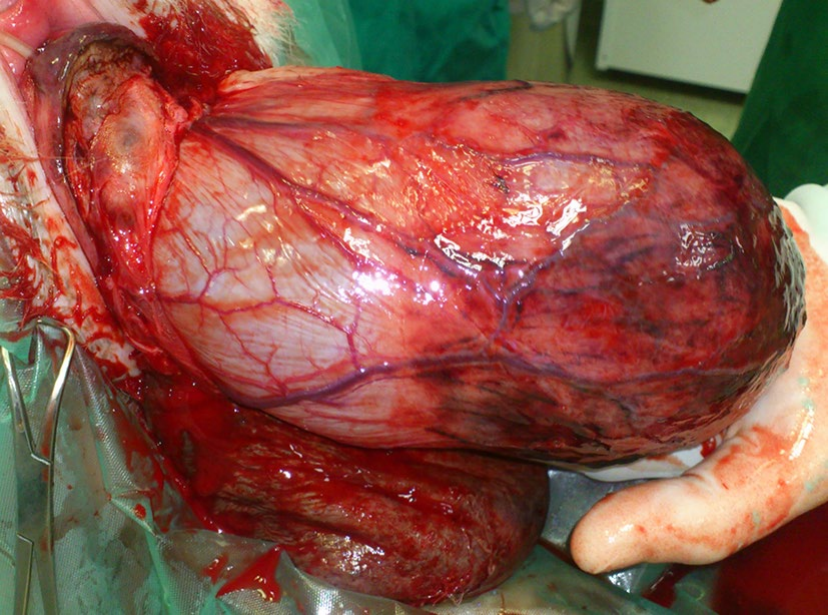
Aspect of the retroflexed bladder after the removal of necrotic vaginal tissue. The middle and cranial regions are locally extensive congested and edematous

The abdominal, vulvar and anal regions were prepared for surgery. The dog was placed on a padded rectal stand and the vulvar region was draped for surgery. Because of significant mucosal injury, resection of the vaginal edematous tissue using electrocautery was performed. The middle and cranial parts of urinary bladder were locally extensively congested and edematous. A well-defined line of demarcation between the normal and affected tissue was visible (Fig. 3). The urine was evacuated from the bladder with a 21G needle and bladder was gently pushed into the pelvic canal through the vulva. The dog was placed in dorsal recumbency, a midline laparotomy was performed and the urinary bladder was carefully repositioned to its normal anatomic position. Opposing areas of the bladder’s serosa and the right abdominal wall peritoneum were abraded by a scalpel and five interrupted sutures of 3–0 polypropylene (Polypropylene; Assut sutures, Switzerland) were placed between the bladder and abdominal wall to hold the urinary bladder and prevent recurrence. The urethral opening was then catheterised without any problem. An ovariohysterectomy was performed after cystopexy.

The rectal mucosa was cleaned with warm isotonic NaCl solution and repositioning was attempted, but unsuccessful despite multiple attempts to gently reduce the prolapse. The tissue viability was questionable because of oedema and necrosis, so rectal resection and anastomosis was judged necessary. The dog was again placed on the padded rectal stand, and the perineal region was prepped and draped for surgery. A test tube was introduced into the rectum to aid in suture placement and prevent fecal contamination during surgery. The prolapsed tissue was resected 2 cm from the anus. The two ends were full thickness anastomosed with a single layer of simple interrupted, synthetic, absorbable monofilament PDS sutures.

Amoxicillin–clavulanic acid (25 mg/kg BW bid; Synulox; Zoetis, UK) and carprofen (2 mg/kg BW bid; Ricarfa, KRKA, București, Romania) were administered after surgery for 6 days.

The dog recovered completely after the surgery. No recurrence of the prolapsed vagina or rectum and no retroflexion of the urinary bladder occurred during 2 months follow-up.

## Conclusions

Vaginal edema in bitches commonly develops in proestrus and estrus and in the first three estrous cycles in younger bitches [6]. This condition must be differentiated from vaginal prolapse, e.g. by digital vaginal exploration. The incidence of prolapsed vagina is higher in large-breed dogs [1, 9], but true vaginal prolapse is extremely rare in dogs and cats [10] and mainly occurs during parturition or shortly after [11]. Because the dog in the study was a young, non-pregnant female and it was not separated by force during breeding, constipation (although not reported) could be a possible predisposing condition as reported in other studies [2, 3].

The median ligament (*lig. vesicae medianum*) and the lateral ligaments (*lig. vesicae laterales*) of the bladder hold the organ in its normal position. The ligaments are made up of double layers of peritoneum separated by intercalated blood vessels, nerves, lymphatics, and adipose tissue, as well as by the ureters, deferent ducts, and vestiges of embryonic structures [12]. In a previous report [7], similar to the case in this study, the authors speculate that excessive tenesmus because of the rectal prolapse resulted in increased intra-abdominal pressure, rupture of the ligaments, caudal displacement of the urinary bladder, and protrusion of the bladder into the vulvar cleft. However, in our case, rectal prolapse was secondary to vaginal prolapse. Therefore, in our case, excessive straining and tenesmus due to the vaginal prolapse and bladder retroflexion may have resulted in disruption of the supporting ligaments of the colon.
